# Supplementary material for: Management of insecticides for use in disease vector control: Lessons from six countries in Asia and the Middle East
Source: PLoS Negl Trop Dis. 2021 Apr 30;15(4):e0009358. doi: 10.1371/journal.pntd.0009358 (PMC8115796; doi:10.1371/journal.pntd.0009358)
Supplement: S1 Appendix — (DOCX) [file pntd.0009358.s001.docx]

**Supporting information**

**S1 Appendix.** Panel of experts consulted in the selected countries.

| **Country** | **Area of expertise** | **Agency** | **Nr. of experts consulted** |
| --- | --- | --- | --- |
| Bangladesh | Health policy | Directorate General of Health Services (DGHS), Ministry of Health & Family Welfare | 1 |
|  | Malaria and dengue control | Malaria and Aedes Transmitted Diseases Control Programme, Directorate General of Health Services, Ministry of Health & Family Welfare | 4 |
|  | Medical entomology | Institute of Epidemiology, Disease Control and Research, Dhaka | 1 |
|  | Medical entomology | Jahangirnagar University, Dhaka | 1 |
|  | Mosquito control operations | Dhaka North City Corporation | 1 |
|  | Mosquito control operations | Dhaka South City Corporation | 1 |
|  | Decentralized health services | Ministry of Local Government, Rural Development Co-Operatives | 1 |
|  | Water and sanitation | Ministry of Local Government, Rural Development Co-Operatives | 1 |
|  | Pesticide registration | Department of Agricultural Extension, Ministry of Agriculture | 1 |
|  | Kala-azar elimination | National Kala-azar Elimination Program, DGHS, Ministry of Health & Family Welfare | 2 |
|  | Insecticide resistance monitoring | International Centre for Diarrhoeal Disease Research-Bangladesh | 2 |
|  | Pest and pesticide management | FAO-Bangladesh | 2 |
|  | Vector-borne disease control | WHO-Bangladesh | 5 |
|  |  |  |  |
| Cambodia | Public health pesticide management | Department of Preventive Medicine Department, Ministry of Health | 2 |
|  | Malaria control | National Malaria Control Programme, National Centre for Parasitology Entomology and Malaria Control | 1 |
|  | Dengue control | National Dengue Control Program, National Centre for Parasitology Entomology and Malaria Control | 1 |
|  | Public health product registration | Department of Drugs and Food, Ministry of Health | 2 |
|  | Pesticide poisoning | Emergency Service, Calmette Hospital, Phnom Penh | 1 |
|  | Pesticide management | FAO-Cambodia | 1 |
|  | Vector-borne disease control | WHO-Cambodia | 4 |
|  |  |  |  |
| Nepal | Vector-borne disease control | Epidemiology and Disease Control Division, Department of Health Services, Ministry of Health and Population | 3 |
|  | Vector control inspection | Epidemiology and Disease Control Division, Department of Health Services, Ministry of Health and Population | 1 |
|  | Pesticide registration | Plant Quarantine and Pesticide Management Centre, Ministry of Agriculture | 2 |
|  | Plant protection | Plant Quarantine and Pesticide Management Centre, Ministry of Agriculture | 1 |
|  | Pesticide retail | Pesticide Entrepreneurs Association, Nepal | 3 |
|  | Pesticide pollution and poisoning | Center for Public Health and Environmental Development (NGO) | 2 |
|  | Entomology | Nepal Agricultural Research Council (NARC) | 3 |
|  | Vector-borne disease control | WHO-Nepal | 4 |
|  |  |  |  |
| Sri Lanka | Health policy | Directorate General (Public Health), Ministry of Health | 1 |
|  | Malaria elimination | Anti-Malaria Campaign, Ministry of Health | 1 |
|  | Malaria entomology | Anti-Malaria Campaign, Ministry of Health | 2 |
|  | Malaria vector control | Regional Malaria Office, Kurunegala | 1 |
|  | Malaria vector control | Regional Malaria Office, Puttalam | 1 |
|  | Dengue control | National Dengue Control Unit, Ministry of Health | 4 |
|  | Dengue entomology | National Dengue Control Unit, Ministry of Health | 1 |
|  | Filariasis control | Anti-Filariasis Campaign, Ministry of Health | 1 |
|  | Personal protection | Environmental and Occupational Health, Ministry of Health | 2 |
|  | Pesticide poisoning | Poison Information Centre, National Hospital, Colombo | 1 |
|  | Pesticide registration | Registrar of Pesticides, Department of Agriculture, Peradeniya | 3 |
|  | Insecticide resistance; dengue vector control | Peradeniya University | 2 |
|  | Dengue outbreak control | Public Health Department, Colombo Municipal Council | 1 |
|  | Dengue vector control | Public Health Department, Colombo Municipal Council | 2 |
|  | Pesticide waste management | Chemical and Hazardous Waste Management Unit, Central Environmental Authority | 2 |
|  | Pesticide distributor | Private sector company | 1 |
|  | Communicable diseases control | WHO-Sri Lanka | 2 |
|  |  |  |  |
| Oman | Public health | WHO-Oman | 2 |
|  | Health policy | Disease Surveillance and Control Directorate, Ministry of Health | 2 |
|  | Medical entomology | Vector Surveillance Section Supervisor, Epidemiological Surveillance Department, Disease Surveillance and Control Directorate, Ministry of Health | 1 |
|  | Vector surveillance | Vector Surveillance Section Supervisor, Epidemiological Surveillance Department, Disease Surveillance and Control Directorate, Ministry of Health | 2 |
|  | Personal protection, pesticide poisoning | Department of Environmental and Occupational Health, Ministry of Health | 3 |
|  | Plant protection | Plant Protection, Ministry of Agriculture and Fisheries | 3 |
|  | Pesticide management | Pesticide Management, Ministry of Agriculture and Fisheries | 1 |
|  | Pesticide registration | Pesticide registration office, Ministry of Agriculture and Fisheries | 3 |
|  | Entomological research | Research Centre, Ministry of Agriculture and Fisheries | 1 |
|  | Pesticide quality control | Pesticides Residue & Quality Control Section, Research Centre, Ministry of Agriculture and Fisheries | 1 |
|  | Entomological research | Research Centre, Ministry of Agriculture and Fisheries | 3 |
|  | Zoonotic diseases | Veterinary Animal Health Department, Ministry of Agriculture and Fisheries | 1 |
|  | Civil society representative | Omani Agricultural Association, Al Suwayq, North Batinah Governorate | 1 |
|  | Mosquito control operations | Directorate General of Health Affairs, Muscat Municipality | 4 |
|  | Pesticide waste management | Waste Management Section and Chemical Classification Section, Ministry of Environment and Climate Affairs | 2 |
|  |  |  |  |
| Vietnam | Environmental health policy | Directorate General, Health Environment Management Agency, Ministry of Health | 1 |
|  | Public health pesticide registration | Environment Health and Chemical Management Division, VIHEMA, Health Environment Management Agency, Ministry of Health | 2 |
|  | Communicable diseases control | WHO-Vietnam | 1 |
|  | Malaria control operations | Department of Planning, National Institute of Malaria, Parasitology and Entomology (NIMPE), Ministry of Health | 1 |
|  | Malaria entomology and vector control | Department of Entomology, National Institute of Malaria, Parasitology and Entomology (NIMPE), Ministry of Health | 1 |
|  | Dengue control | Division of Communicable Disease Control, General Department of Preventive Medicine, Ministry of Health | 1 |
|  | Dengue vector control | Dengue Prevention and Control Program, General Department of Preventive Medicine, Ministry of Health | 1 |
