# Supplementary material for: Management of insecticides for use in disease vector control: Lessons from six countries in Asia and the Middle East
Source: PLoS Negl Trop Dis. 2021 Apr 30;15(4):e0009358. doi: 10.1371/journal.pntd.0009358 (PMC8115796; doi:10.1371/journal.pntd.0009358)
Supplement: S2 Appendix — (DOCX) [file pntd.0009358.s002.docx]

**Supporting information**

**S2 Appendix.** List of country reports with availability from third party sources.

Bangladesh:

Report: Public health pesticide management in Bangladesh: Situational analysis, needs assessment and action plan, October 2019. By H. van den Berg. Unpublished Report, World Health Organization, 27p.

Available from: World Health Organization, Country Office for Bangladesh, Address: United House (GF to 3rd Floor), 10 Gulshan Avenue, Gulshan 1, Dhaka-1212, Bangladesh. E-mail: sebanregistry@who.int

Nepal:

Report: Public health pesticide management in Nepal: Situational analysis and needs assessment. November 2019. By H. van den Berg. Unpublished Report, World Health Organization, 20p.

Available from: WHO Representative Office, WHO Nepal, United Nations House, Pulchowk, Lalitpur, Kathmandu. E-mail: senepwr@who.int

Sri Lanka:

Report: Public health pesticide management in Sri Lanka: Situational analysis, needs assessment and action plan. August 2019. By H. van den Berg. Unpublished Report, World Health Organization, 27p.

Available from: WHO Representative Office, Country Office for Sri Lanka, 5 Anderson Road, Colombo 05. Email: sesrlregistry@who.int

Cambodia:

Report: Public health pesticide management in Cambodia: Recent progress, needs assessment and action plan. December 2019. By H. van den Berg. Unpublished Report, World Health Organization, 27p.

Available from: WHO Representative Office, 1st Floor No. 61-64, Preah Norodom Blvd. (corner St. 306), Sangkat Boeung Keng Kang I Khan, Chamkamorn, Phnom Penh, Cambodia. Email: wpkhmwr@who.int

Vietnam:

Report: Public health pesticide management in Viet Nam: Situational analysis, needs assessment and action plan. November 2019. By H. van den Berg. Unpublished Report, World Health Organization, 22p.

Available from: WHO Representative Office, 304 Kim Ma Street, Ha Noi, Viet Nam. Email: wpvnmwr@who.int

Oman:

Report: Public health pesticide management in the Sultanate of Oman: Recent progress, needs assessment and action plan. September 2019. By H. van den Berg. Unpublished Report, World Health Organization, 27p.

Available from: WHO Representative Office, PO Box 1889, Ruwi, Muscat 112, Oman. Email: boukamelr@who.int
