# Supplementary material for: Management of insecticides for use in disease vector control: Lessons from six countries in Asia and the Middle East
Source: PLoS Negl Trop Dis. 2021 Apr 30;15(4):e0009358. doi: 10.1371/journal.pntd.0009358 (PMC8115796; doi:10.1371/journal.pntd.0009358)
Supplement: Attachment — Submitted filename: PNTD-D-20-01641_reviewed.pdf [file pntd.0009358.s004.pdf]

PLOS Neglected Tropical Diseases

Management of insecticides for use in disease vector control: Lessons from six countries in Asia and the Middle East

--Manuscript Draft--

**Manuscript Number:**	PNTD-D-20-01641
**Full Title:**	Management of insecticides for use in disease vector control: Lessons from six countries in Asia and the Middle East
**Short Title:**	Management of vector control insecticides
**Article Type:**	Research Article
**Keywords:**	neglected tropical diseases, vector-borne diseases; malaria; leishmaniasis; dengue; disease endemic countries; pesticide life cycle management; insecticide resistance; entomological capacity; environmental risks; risk to human health; regulatory control.
**Abstract:**	Interventions to control the vectors of human diseases, notably malaria, leishmaniasis and dengue, have relied mainly on the action of chemical insecticides. However, concerns have been raised regarding the management of insecticides in vector-borne disease-endemic countries. Our study aimed to analyze how vector control insecticides are managed in selected countries to extract lessons learned.<br>A qualitative analysis of the situation of vector control insecticides management was conducted in six countries. Multi-stakeholder meetings and key informer interviews were conducted on aspects covering the pesticide lifecycle. Findings were compared and synthesized to extract lessons learned. Centrally executed guidelines and standards on the management of insecticides offered direction and control in most malaria programs, but were largely lacking from decentralized dengue programs, where practices of procurement, application, safety, storage, and disposal were variable between districts. Decentralized programs were better at facilitating participation of stakeholders and local communities and securing financing from local budgets. However, little coordination existed between malaria, leishmaniasis and dengue programs within countries. Entomological capacity was concentrated in malaria programs at central level, while dengue programs were missing out on expertise. Monitoring systems for insecticide resistance in malaria vectors were rarely used for dengue vectors. Strategies for insecticide resistance management, where present, did not extend across programs or sectors in most countries. Dengue programs in most countries continued to rely on space spraying which, considering the realities on the ground, call for revision of international guidelines.<br>Vector control programs in the selected countries were confronted with critical shortcomings in the procurement, application, safety measures, storage, and disposal of vector control insecticides, with implications for the efficiency, effectiveness, and safety of vector control. Further international support is needed to assist countries in situation analysis, action planning and development of national guidelines on vector control insecticide management.
**Additional Information:**	
**Question**	**Response**
**Financial Disclosure**<br><br>Enter a financial disclosure statement that describes the sources of funding for the work included in this submission. Review the submission guidelines for detailed requirements. View published research articles from *PLOS NTDs* for specific examples.<br><br>This statement is required for submission	The work was supported by the Bill & Melinda Gates Foundation, Seattle, WA, as part of the Innovation-2-Impact project (grant number OPP1133139) awarded to the World Health Organization (RV and RSY). The funders had no role in study design, data collection and analysis, decision to publish, or preparation of the manuscript.

Powered by Editorial Manager® and ProduXion Manager® from Aries Systems Corporation

and **will appear in the published article** if the submission is accepted. Please make sure it is accurate.<br><br>**Unfunded studies**<br><br>Enter: *The author(s) received no specific funding for this work.*<br><br>**Funded studies**<br><br>Enter a statement with the following details:<br>• Initials of the authors who received each award<br>• Grant numbers awarded to each author<br>• The full name of each funder<br>• URL of each funder website<br>• Did the sponsors or funders play any role in the study design, data collection and analysis, decision to publish, or preparation of the manuscript?<br>• **NO** - Include this sentence at the end of your statement: *The funders had no role in study design, data collection and analysis, decision to publish, or preparation of the manuscript.*<br>• **YES** - Specify the role(s) played.<br><br>* typeset	
**Competing Interests**<br><br>Use the instructions below to enter a competing interest statement for this submission. On behalf of all authors, disclose any competing interests that could be perceived to bias this work—acknowledging all financial support and any other relevant financial or non-financial competing interests.<br><br>This statement **will appear in the published article** if the submission is accepted. Please make sure it is	The authors have declared that no competing interests exist.

accurate. View published research articles from *PLOS NTDs* for specific examples.<br><br>**NO authors have competing interests**<br><br>Enter: *The authors have declared that no competing interests exist.*<br><br>**Authors with competing interests**<br><br>Enter competing interest details beginning with this statement:<br><br>*I have read the journal's policy and the authors of this manuscript have the following competing interests: [insert competing interests here]*<br><br>* typeset	
**Data Availability**<br><br>Authors are required to make all data underlying the findings described fully available, without restriction, and from the time of publication. PLOS allows rare exceptions to address legal and ethical concerns. See the PLOS Data Policy and FAQ for detailed information.<br><br>A Data Availability Statement describing where the data can be found is required at submission. Your answers to this question constitute the Data Availability Statement and **will be published in the article**, if accepted.	No - some restrictions will apply

Powered by Editorial Manager® and ProduXion Manager® from Aries Systems Corporation

**Important:** Stating 'data available on request from the author' is not sufficient. If your data are only available upon request, select 'No' for the first question and explain your exceptional situation in the text box.<br><br>Do the authors confirm that all data underlying the findings described in their manuscript are fully available without restriction?	
**Describe where the data may be found in full sentences. If you are copying our sample text, replace any instances of XXX with the appropriate details.**<br><br>• If the data are **held or will be held in a public repository**, include URLs, accession numbers or DOIs. If this information will only be available after acceptance, indicate this by ticking the box below. For example: *All XXX files are available from the XXX database (accession number(s) XXX, XXX.*).<br>• If the data are all contained **within the manuscript and/or Supporting Information files**, enter the following: *All relevant data are within the manuscript and its Supporting Information files.*<br>• If neither of these applies but you are able to provide **details of access elsewhere**, with or without limitations, please do so. For example:<br><br>*Data cannot be shared publicly because of [XXX]. Data are available from the XXX Institutional Data Access / Ethics Committee (contact via XXX) for researchers who meet the criteria for access to confidential data.*<br><br>*The data underlying the results*	All relevant data are within the manuscript and its Supporting Information files.

presented in the study are available from (include the name of the third party and contact information or URL).<br>• This text is appropriate if the data are owned by a third party and authors do not have permission to share the data.<br><br>* typeset	
Additional data availability information:	

Powered by Editorial Manager® and ProduXion Manager® from Aries Systems Corporation

Manuscript

Click here to access/download;Manuscript;MS Vector control insecticide management.docx

PLOS Neglected Tropical Diseases (research)

Management of insecticides for use in disease vector control: Lessons from six countries in Asia and the Middle East

Henk van den Berg[1], Raman Velayudhan[2], Rajpal S. Yadav[2*]

[1]Laboratory of Entomology, Wageningen University, Wageningen, the Netherlands

[2]Veterinary Public Health, Vector Control and Environment Unit, Department of Control of Neglected Tropical Diseases, World Health Organization, Geneva, Switzerland

*Corresponding author

E-mail: yadavraj@who.int (RSY)

ORCID nr. H. van den Berg: 0000-0002-9983-638X

ORCID nr. R.S. Yadav: 0000-0001-8264-1204

Short title: Management of vector control insecticides

Funding: The work was supported by the Bill & Melinda Gates Foundation, Seattle, WA, as part of the Innovation-2-Impact project (grant number OPP1133139) awarded to the World Health Organization (RV and RSY). The funders had no role in study design, data collection and analysis, decision to publish, or preparation of the manuscript.

Competing interests: The authors have declared that no competing interests exist.

Author Contributions

Conceptualization: Henk van den Berg, Rajpal S. Yadav, Raman Velayudhan. **Data curation**: Henk van den Berg. **Formal analysis**: Henk van den Berg. **Funding acquisition**: Rajpal S. Yadav, Raman Velayudhan. **Investigation**: Henk van den Berg. **Methodology**: Henk van den Berg. **Project administration**: Rajpal S. Yadav. **Supervision**: Rajpal S. Yadav, Raman Velayudhan. **Validation**: Henk van den Berg, Rajpal S. Yadav. **Visualization**: Henk van den Berg. **Writing – original draft**: Henk van den Berg. **Writing – review & editing**: Henk van den Berg, Raman Velayudhan, Rajpal S. Yadav.

Line 50-55 You are referring from line 50 to 55 about malaria and dengue only, what about leishmaniasis programs? There are plenty of reports stating that the effect of insecticides were not as effective due to lack of proper training for the application of insecticides. Same comment applies for line 69 to 73. You should discuss as well leishmaniasis control programs when compared to dengue and malaria.

Abstract

Interventions to control the vectors of human diseases, notably malaria, leishmaniasis and dengue, have relied mainly on the action of chemical insecticides. However, concerns have been raised regarding the management of insecticides in vector-borne disease-endemic countries. Our study aimed to analyze how vector control insecticides are managed in selected countries to extract lessons learned.

A qualitative analysis of the situation of vector control insecticides management was conducted in six countries. Multi-stakeholder meetings and key informer interviews were conducted on aspects covering the pesticide lifecycle. Findings were compared and synthesized to extract lessons learned. Centrally executed guidelines and standards on the management of insecticides offered direction and control in most malaria programs, but were largely lacking from decentralized dengue programs, where practices of procurement, application, safety, storage, and disposal were variable between districts. Decentralized programs were better at facilitating participation of stakeholders and local communities and securing financing from local budgets. However, little coordination existed between malaria, leishmaniasis and dengue programs within countries. Entomological capacity was concentrated in malaria programs at central level, while dengue programs were missing out on expertise. Monitoring systems for insecticide resistance in malaria vectors were rarely used for dengue vectors. Strategies for insecticide resistance management, where present, did not extend across programs or sectors in most countries. Dengue programs in most countries continued to rely on space spraying which, considering the realities on the ground, call for revision of international guidelines.

Vector control programs in the selected countries were confronted with critical shortcomings in the procurement, application, safety measures, storage, and disposal of vector control insecticides, with implications for the efficiency, effectiveness, and safety of vector control.

Further international support is needed to assist countries in situation analysis, action planning and development of national guidelines on vector control insecticide management.

Author summary

Vector-borne diseases such as dengue, malaria and leishmaniasis are transmitted by insect vectors. Transmission can be interrupted through vector control. Chemical insecticides are the mainstay for controlling these insect vectors. However, the use of chemicals also introduces risks to health and the environment and may lead to insecticide resistance. Hence, proper management of those insecticides is critical. To find out how the insecticides used for vector control are being managed, the authors conducted investigations in six countries in Asia and the Middle East. They found that the practices of insecticide procurement, application, storage, and disposal depended on how a program is organized. Dengue programs were operated in a decentralized manner and, consequently, lacked coordination through guidelines and standards on best practices. Also, coordination between malaria and dengue programs within countries was minimal, and expertise needed to guide decisions on vector control and to monitor insecticide resistance was in short supply. The identified shortcomings in how vector control insecticides are managed likely affected the efficiency, effectiveness, and safety of vector control operations.

Introduction

Vector-borne diseases, including malaria, dengue, lymphatic filariasis, schistosomiasis, chikungunya, onchocerciasis, Chagas disease, leishmaniasis, Zika virus disease, yellow fever and Japanese encephalitis, account for an estimated 17% of the global burden of communicable diseases [1, 2], and over half of the global population lives in areas at risk of two or more of the major vector-borne diseases [3]. Notwithstanding the diversity of pathogenic agents, their epidemiology, burden and available treatments, all vector-borne diseases have in common that

their transmission through mosquito, blackfly, triatomine, sand fly, or snail vectors can be interrupted by vector control. Indeed, vector control interventions have demonstrated their critical importance for the control and elimination of most vector-borne diseases [4-8].

Vector control interventions have relied to a large extent on the action of chemical insecticides, applied to surfaces, water bodies, as aerial application, or impregnated in long-lasting netting materials [9-11]. The use of non-chemical options of vector control, such as environmental management or the use of microbial products, remains marginal for a disease like malaria [12], even though the need for alternative methods has been emphasized [4, 13-15]. Moreover, the optimal implementation and monitoring of vector control interventions continues to be a major challenge in many countries [16-19].

Line 95-96- Changed to the following: Their strategy is not disease-specific but seeks to .... (as it sounds repetitive)

The World Health Organization (WHO) recently launched the Global Vector Control Response 2017-2030 [1, 20] to advance the cause of integrated vector management [21, 22]. This strategy is not a disease-specific strategy but seeks to strengthen the structures, capacities, and linkages needed to boost the sustainable role of vector control in achieving targets for control and elimination of all major vector-borne diseases. Amongst others, it calls for the improved deployment of available vector control tools, the mainstay of which have been insecticidal tools, by basing selections on efficacy data, with efficient delivery of quality products, with capacity to manage insecticide resistance, and with minimized adverse effects on health and the environment. Line 95-96- Missing reference for line 98-102

However, results from a recent study on pesticide lifecycle management showed shortcomings in many countries across regions in terms of insecticide resistance monitoring, procurement procedures, applicator safety measures, storage conditions, and waste disposal [23]. Moreover, regulatory control and quality control of public health pesticides (including vector control insecticides) were a concern, particularly in low-income countries and small countries. These

Line 95-96- Missing reference for line 106-107

shortcomings in pesticide lifecycle management could compromise the efficient and efficacious use of vector control insecticides, while posing risks to health and the environment.

Detailed studies at country level can provide more insight into the situation of vector control insecticide management, the causes of deficiencies, the context in which decisions have been made, and the opportunities for structural improvements. We conducted an analysis of the situation of vector control insecticides management in selected countries intended to assist these countries in needs assessment and action planning. The objective of this study was to compare and synthesize the analyses from the six countries to extract lessons learned.

Methods

Country selection

The selection of countries was conducted based on inquiry from WHO's regional offices (except for the regional office for Europe) about country interest in situation analysis and action planning on public health pesticide management. Following the response from the regional offices, a decision was made to concentrate the study on countries in Asia and the Middle East. After verification of country interest through direct communication with WHO country offices, six countries agreed to participate in the activities and all six were selected for study. The selected countries were Bangladesh, Cambodia, Nepal, Oman, Sri Lanka, and Vietnam (Fig 1).

Line 123-124: Why was India not included as selected country in your study? India is highly endemic for Dengue, leishmaniasis and malaria and their major vector control program is through the use of insecticides. I think it would bring impact to the manuscript to at add or discussed the current situation there.

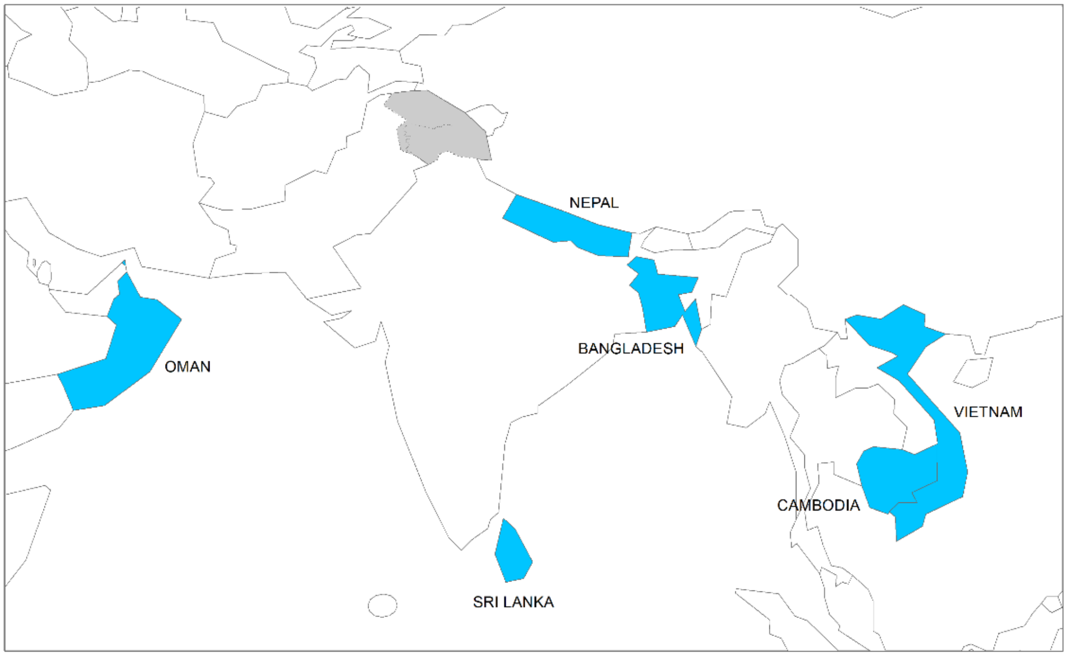

The boundaries and names shown and the designations used on this map do not imply the expression of any opinion whatsoever on the part of the World Health Organization concerning the legal status of any country, territory, city or area or of its authorities, or concerning the delimitation of its frontiers or boundaries. Dotted and dashed lines on maps represent approximate border lines for which there may not yet be full agreement.

Fig 1. Map showing countries selected for the study.

To verify whether the selected countries were representative for their regions, we examined the data from a separate global survey conducted in 2018 [23], in which fifteen quality indicators on vector control insecticide management were recorded for 31 countries in Asia and the Middle East, with an average score of 52% (higher is better). By comparison, our six selected countries scored an average of 40%, suggesting that the selected countries had slightly poorer conditions of vector control insecticide management than other countries in their regions.

Data collection

Preparatory consultations were held with focal points in each country to identify, and arrange meetings with, the main experts in the management of public health pesticides in each country. A list of the panel of experts in each country, with area of expertise and agency, are available in the Supplementary Information (S1 Table). Study visits to each selected country were made by one of us (HB) over the period August-December 2019. The methods in each country

included interview meetings with one or more key staff from each identified stakeholder to gather information and data and, in Bangladesh, Nepal and Oman, a multi-stakeholder meeting, to discuss strengths, weaknesses and needs. Depending on the country, stakeholders included ministries of health, agriculture, local government and environment, vector-borne disease program offices, pesticide registration offices, pesticide analysis laboratories, municipalities, civil society organizations, the private sector, and academic institutions. Visits were made to pesticide laboratories, entomology laboratories in all countries, and to field spray teams in Bangladesh, Oman, and Sri Lanka. The scope of the assessment included technical, organizational, and institutional aspects of the following topics: pesticide regulatory control, pesticide procurement, insecticide application for vector control, insecticide resistance management, safety measures, pesticide storage, pesticide disposal, and waste management. A list of questions was prepared for each topic as a guide during the interviews, and the responses were documented. Institutional and organizational aspects regarding each topic (e.g. regarding coordination, capacity) were explored through discussion. Data on disease cases were obtained from a national database in each country. For each country we documented the findings in a country report. We consulted additional data and information sources, including national guideline or strategic documents, scientific reports, regional workshop reports and published literature to provide verification and insight into the subject matter. Where claims could be verified, we have noted this is the text.

Data interpretation

The outcomes of the six country reports were reviewed to identify themes of interest. Excluded were issues referring to pesticides other than vector control insecticides. The identified themes were: organizational aspects of vector control, coordination between programs, inter-sectoral coordination, entomological capacity, insecticide resistance management, pesticide regulatory control, pesticide procurement, application methods and safety measures, and pesticide storage

and disposal. For each theme, the issues at hand were synthesized to extract relevant lessons learned across countries. Where possible, the information received through verbal communication was verified through review of available documentation and direct observation. Countries were anonymized for information that was considered potentially sensitive.

Results

Vector control programs

The number of vector (or nuisance pest) control programs varied from two to four per country (Table 1). Malaria vector control programs existed in all countries, in phases ranging from malaria elimination to prevention-of-reintroduction of malaria. Malaria cases were approaching elimination in Bangladesh, Cambodia, Nepal, and Vietnam; and were zero or almost zero in Oman and Sri Lanka. *Plasmodium vivax* was dominant among malaria cases in Cambodia and Nepal whilst *P. falciparum* was dominant among cases in Bangladesh and Vietnam. Bangladesh and Nepal had vector control programs on elimination of visceral leishmaniasis ('kala-azar'),

Text

Line 178. The six countries had..

with relatively small numbers of annual cases reported. Six countries had vector control programs on emergency response and control of dengue, showing a recent increase in the number of cases, particularly in Bangladesh, Cambodia, Sri Lanka, and Vietnam.

In the selected countries, programs on elimination of malaria and kala-azar depended primarily on insecticide-treated bed nets and indoor residual spraying for vector control (Table 1), using pyrethroids for both methods, despite partly outdoor-biting behavior of vectors and despite the emergence of pyrethroid resistance. Indoor residual spraying was used for reduction of the

Line 183-184. Missing references of pyrethroid resistance and outdoor-bitting behavior of the vectors

malaria burden or for emergency control where sporadic malaria cases were found. Insecticidal hammock nets and topical repellents were distributed as additional tools to protect forest-goers against malaria in Cambodia and Vietnam, whilst larviciding was used in receptive sites for prevention-of-reintroduction of malaria in Sri Lanka and Oman.

Add references on the actors column in Table 1 for each of the vector control programs. .

Table 1. The programs with vector control component in the selected countries.

Country	Vector-borne disease or pest targeted	Cases[a]	Control stage[b]	Vector control tools[c]	Organizational structure[d]	Actors
Bangladesh	Malaria	10,500	2	3,4	C	Malaria program
	Visceral leishmaniasis	144	2	3,4	C	Kala-azar program
	Dengue	90,000	3,4	1,2,5	D	Municipalities, malaria/dengue unit
	Nuisance mosquitoes	n/a	4	2,5	D	Municipalities
Cambodia	Malaria	25,502	2	3,7	C	Malaria program, PMI
	Dengue	67,436	3,4	1,2,5	D	Dengue program, provinces, districts
Nepal	Malaria	1,065	2	3,4	C	Malaria Program
	Visceral leishmaniasis	169	2	4	C	Kala-azar program
	Dengue	14,000	3,4	1	D	Dengue program, municipalities
Sri Lanka	Malaria	1	1	2,3,4	C/D	Malaria program
	Dengue	99,120	3,4	1,2,4,5	D/C	Dengue program, municipalities
Oman	Malaria	0	1	2,5	C	Ministry of Health
	Dengue	59	3,4	2,5,6	C/D	Ministry of Health, municipalities
	Nuisance mosquitoes	n/a	4	2,5	D	Municipalities
Vietnam	Malaria	5,000	2	3,4,7	C	Malaria program
	Dengue, chikungunya	250,000	3,4	1,2,5	D/C	Dengue program, municipalities

[a] Annual number of cases in 2019 or, where unavailable, in 2018; n/a signifies 'not applicable'

[b] 1, Prevention-of-reintroduction; 2, elimination; 3, emergency response; 4, control

[c] 1, Source reduction or environmental management; 2, larviciding; 3, long-lasting insecticidal nets (LLIN) or long-lasting insecticidal hammock nets; 4, indoor residual spraying (IRS); 5, space spraying (fogging); 6, peri-focal residual spraying around breeding sites; 7, topical repellents

[d] C, centralized; D, decentralized

In dengue control, 'source reduction', which is the removal or coverage of water-holding containers in which dengue vectors breed, was promoted as core intervention in all endemic countries. Space spraying to kill flying vectors, and larviciding to kill the aquatic immature stages, were targeted at dengue outbreak sites in five countries (Table 1); main insecticides used were the organophosphate temephos for larviciding and pyrethroids for space spraying. Space spraying was conducted mainly outdoors, but in addition in Sri Lanka and Vietnam, ultra-low volume space spraying was applied indoors during an emergency response. In Nepal, space spraying was discouraged by national health authorities because of the notion that spray operations would result in widespread insecticide use and reduce the active role of communities in source reduction activities. In Bangladesh and Oman, space spraying was routinely carried out against nuisance mosquitoes in urban environments.

Organizational structures for vector control

The way in which vector control is organized at country level has implications for pesticide management. Some programs in the selected countries used a centralized mechanism, in which planning, and financing of vector control were directed by health agencies at national level. Other programs adopted a decentralized or partially decentralized mechanism, in which elements of planning, financing and implementation were delegated to health authorities at the provincial, district or municipal level. Centralized and decentralized programs were operated in parallel within countries.

Centralized programs included those for elimination and prevention-of-reintroduction of malaria, which were generally spearheaded by the national malaria control program authorities – in keeping with the management structure adopted during the first global malaria eradication program [24]. In two countries, components of malaria program management had been delegated to the provinces and districts. The core vector control interventions (insecticide-treated bed nets and indoor residual spraying) aimed for universal coverage of targeted communities, which may have benefited from centralized planning and resource allocation. Also, epidemiological and entomological surveillance, where available, were mostly planned at the central level. The programs on elimination of visceral leishmaniasis followed a centralized mechanism like for malaria elimination.

Conversely, dengue programs were mostly decentralized to district or municipal health offices. The core intervention, source reduction, depended on the active participation of communities, including schools and workplaces. Hence, source reduction may have benefited from a decentralized setting. Space spraying and larviciding were used in response to dengue outbreaks by district or municipal health offices in five countries. In Bangladesh, dengue outbreak response was taken up by municipal health authorities, tasked to the spray teams responsible

for routine control of nuisance mosquitoes. In two countries, the national health authorities assisted smaller municipalities outside of the capital city in their dengue control activities.

Decentralized dengue programs in five countries had a central dengue unit, tasked with strategic development and training. Four countries had developed national guidelines on dengue control. Vietnam established a centralized online reporting system for dengue-case data from hospitals and clinics, which was used by sub-national institutions to advice district health centers on emergency response action. Other countries lacked a national online reporting system and, thus, the central unit did not advice the districts on dengue response action. Financing of dengue control activities, including for training and insecticide procurement, was primarily through budget allocation at district or municipal level according to local priority setting, which reportedly resulted in a variable quality of training and operations. In Vietnam and Sri Lanka, the central level co-financed the districts, by contributing to insecticide procurement or by supporting dengue control.

Centralized mechanisms aided vector control on several fronts. National policies and strategies for the control and elimination of malaria provided direction to program activities. National guidelines, certified training, and a central mechanism for insecticide procurement, were critical to the quality and safety of implementation of vector control and pesticide management but these elements were largely absent from dengue programs. Moreover, in Sri Lanka, centralization served to protect national interests concerning the prevention-of-reintroduction of malaria, a subject likely to be undervalued in decentralized priority setting.

Decentralization supported vector control in other ways. The participation of multiple stakeholders and local communities in the prevention of disease transmission, as envisaged in the Global Vector Control Response, was generally easier to achieve at district or municipal level as compared to the central level, because of shorter communication linkages and closer proximity to the field level. For example, during the 2019-dengue outbreak in Dhaka,

Bangladesh, city health departments worked side-by-side with the city's waste management department and with schools to reduce potential breeding sites of *Aedes aegypti* mosquitoes. Conversely, at national level, progress to engage sectors other than the health sector in vector control has been slow or absent in the selected countries. Emergency response action is potentially quicker in a decentralized setting [17], as was found in Colombo Municipal Council, Sri Lanka, where a system of technical experts (epidemiologist, entomologist and pest control officer), case reporting and mobile rapid-response teams reportedly enabled vector control response action within 24 hours of detection of case locations. Furthermore, decentralization improved the prospects for sustained vector control operations because the support for dengue control came from district or municipal budget allocation with little or no external funding. By comparison, centralized programs on malaria elimination in the selected countries faced the uncertainty of continued support for operations after the external donor funding, on which these programs depended, will terminate (Table 2).

Table 2. Lessons and conclusions from the case examples regarding nine themes.

	Theme	Lesson or conclusion
1	Organizational structures for vector control	Some program components benefit from centralization whereas other components benefit from decentralization, suggesting that a mix is optimal for improvement of the efficiency, quality, safety, and sustainability of vector control operations
2	Coordination between vector control programs	The sharing of expertise, equipment and infrastructure between vector-borne disease programs has clear benefits, particularly in settings with a declining trend in malaria versus an increasing trend in dengue. In general, it is not a good solution to simply merge programs, with the intention to reduce the human resources allocated to them
3	Inter-sectoral coordination	Establishing intersectoral collaboration on vector control proved to be difficult, but there are indications that dengue control is a viable entry point, because dengue control depends on the participation of partners at local level, where communication linkages are relatively straightforward
4	Entomological capacity	Enhancement of entomological capacity is most urgent in decentralized dengue programs, to guide local decisions on vector control, but the demand for entomologists is not being met by tertiary education systems
5	Insecticide resistance management	In malaria control, investments in insecticide resistance monitoring systems have not been accompanied by commensurate support for managing resistance effectively or making alternative products and methods available. In dengue control, a lack of resistance management obstructs the efficacious use of insecticides and poses unnecessary risks to health and the environment

6	Pesticide regulatory control	Having a single pesticide registration office has clear advantages for harmonizing procedures and standards across pesticide groups but requires strong linkages between the health and agricultural sectors
7	Pesticide procurement	Central coordination over pesticide procurement has been lacking in decentralized dengue control programs, with possible implications for vector control
8	Application methods and safety measures	With a variety of actors involved in vector control (programs, districts, municipalities), it is critical to strengthen and harmonize application methods and worker safety precautions through the development of national guidelines and protocols
9	Pesticide storage and disposal	Practices of storage and disposal of vector control insecticides in districts and municipalities benefit from national guidelines, standard protocols, training and investment

Coordination between vector control programs

It has been advocated that countries establish coordination between their vector control programs because of potential efficiency gains through the sharing of information, infrastructure and human resources [21]. Oman and Nepal had a national-level vector control unit overseeing all vector-borne diseases. In the case of Oman, this unit coordinated surveillance and outbreak control activities in a situation with only sporadic cases of vector-borne diseases. In Nepal, however, the small national unit with inadequate technical capacity was tasked with coordination of sizable programs for malaria, kala-azar, and dengue.

In other countries, separate national programs existed on malaria and dengue, each with their own technical support. In Cambodia and Vietnam, the malaria program and dengue program operated mostly in isolation, except that in Vietnam, the malaria program provided the dengue program with technical support on vector surveillance, insecticide susceptibility testing and efficacy testing. In Sri Lanka, the program for prevention-of-reintroduction of malaria and the dengue program cooperated at the district level, where local malaria teams shared their entomological expertise with recently-appointed dengue teams and assisted them in the vector control response to dengue outbreaks. Even though malaria has been eliminated in Sri Lanka, the malaria program intended to maintain its separate structure and capacity for vector control,

because merging with the decentralized dengue program would risk that the national importance of prevention-of-reintroduction of malaria would be undervalued in district resource allocation. Bangladesh did not have a separate dengue program but, in response to recent increases in dengue cases, the malaria program had incorporated the control of *Aedes*-transmitted diseases, thus aiding coordination between malaria and dengue.

Hence, two countries had a central coordination unit covering all vector-borne diseases but lacking specific capacity for each disease. Two countries had separate vector control programs which operated mostly in isolation from each other. In two other countries, the expertise, infrastructure, and equipment available in the malaria program were used to assist the less-resourced dengue program in vector surveillance and control. All countries experienced a declining trend in malaria versus an increasing trend in dengue. Hence, it will be vital for countries to ensure continuity of the available entomological expertise and resources at the national level and district level (Table 2).

Inter-sectoral coordination

Sectors outside the health sector have a potential role in vector control, for example, in pesticide management, drainage or irrigation management, waste management or community mobilization. It has been advocated that countries establish a multi-sectoral committee as first step towards involving other sectors in integrated vector management [1, 21]. However, Cambodia and Vietnam did not establish intersectoral linkages or a committee on integrated vector management, whilst Bangladesh and Nepal were planning to establish a committee.

In Oman, earlier attempts in 2007 and 2009 to establish an integrated vector management committee failed, reportedly due to a lack of representation by senior-level decision-makers. In 2017, the committee was re-established, this time with senior-level representation from the implicated ministries, and with a technical task force under its wing. The revived committee demonstrated its value during the 2018-dengue outbreak in Oman when it provided vital

leadership to multiple actors, and successfully secured funds from cabinet for emergency response action. Similarly, in Sri Lanka, a multisectoral committee has since been instrumental in the mobilization of communities and stakeholders in source reduction activities for dengue control, but the scope did not extent to malaria vectors (Table 2).

Entomological capacity

Skilled entomologists (i.e. public health practitioners with basic knowledge about vector biology and epidemiology) are essential for any program with a vector surveillance or vector control component. The number of entomologists ranged from zero to four per vector control program. Nepal had no entomologist was in place for any of the vector control programs on malaria, dengue or kala-azar, and local technicians were absent under the recently federalized government structure. In other countries, entomologists were mainly positioned within malaria programs, mostly at central offices and with assistance by technicians at sub-national or district level. In some countries, however, health authorities expressed concern about the continuity of entomological capacity once malaria has been eliminated.

A critical shortage of entomological capacity was apparent in most dengue programs and in the kala-azar programs. In dengue programs, no entomologists were placed at the district or municipal level, which was the level at which decisions were made on insecticide products, and on the methods, timing and targeting of vector control. An exception was Colombo Municipal Council, Sri Lanka, where an entomologist was present. None of the countries had recently completed a vector control needs assessment [25], but Nepal, Oman and Bangladesh were planning one. Possibilities for tertiary education on medical entomology were absent or limited in the selected countries, suggesting the need for new curricula. Consequently, there was a shortage of graduated entomologists available for recruitment by programs. For example, in Nepal, a vacancy for medical entomologist could reportedly not be filled because qualified candidates were lacking (Table 2).

Insecticide resistance management

Monitoring to detect evidence of insecticide resistance and management to delay development of insecticide resistance have implications for the registration, procurement, and efficacious use of insecticide products.

Malaria programs in the selected countries had systems in place for monitoring insecticide resistance, using the WHO susceptibility test on *Anopheles* spp. collected from sentinel sites. One exception was Nepal, where monitoring of insecticide susceptibility was not carried out and where no recent data were available.

Different stages of managing insecticide resistance in malaria vectors were evident in the selected countries. In Bangladesh, the malaria program had been using deltamethrin for indoor residual spraying for several years and had not added other insecticide options to its arsenal, despite evidence of resistance in part of the sentinel sites. In Nepal, three pyrethroid insecticides were rotated in three-year cycles, but without being informed by insecticide resistance monitoring. Sri Lanka has had a long history using a rotational and mosaic-patterned use of products of insecticides, based on annual susceptibility data from sentinel sites. However, in Vietnam and Sri Lanka, pyrethroids were the only options for rotations in indoor residual spraying, whilst pyrethroids were also being used in insecticide-treated bed nets. In most cases, insecticide resistance is expected to develop against all compounds, in this case all pyrethroids, that share a common mode of action [26]. Hence, even where insecticide resistance monitoring was in place, the data were not optimally used to manage managing resistance or to make alternative products available.

Dengue programs lacked routine monitoring of insecticide resistance in five countries. One exception was Sri Lanka, where susceptibility tests were performed at central and decentralized levels. In Vietnam, the dengue program was supported in susceptibility testing of *Ae. aegypti* by the malaria program, whereas Oman was in the process of including *Ae. aegypti* in routine

susceptibility testing. Data available from Bangladesh, Cambodia, Sri Lanka, and Vietnam indicated high to very high levels of resistance in *Ae. aegypti* adults to pyrethroids and, in some cases, to the organophosphate malathion [27-29]. Despite high levels of resistance, dengue programs continued to use pyrethroids for space spraying, due to a lack of acceptable alternative products or methods for emergency control. Cambodia reported widespread resistance in *Ae. aegypti* larvae to the organophosphorus larvicide temephos [30]. In response, a 2019-pilot was conducted with the biological alternative *Bacillus thuringiensis israelensis*.

Nuisance mosquitoes, particularly *Culex quinquefasciatus*, were a main target for routine insecticidal application by municipalities in Bangladesh and Oman. *Cx. quinquefasciatus* exhibited very high levels of resistance to almost all insecticides tested in both countries, but routine spraying and product selection remained unaltered, and likely accelerated resistance development in *Ae. aegypti*. The kala-azar programs in Bangladesh and Nepal did not have adequate monitoring or management of insecticide resistance of sandfly vectors in place.

Several countries were in the process of developing strategies for insecticide resistance management in conjunction with the agricultural sector, to coordinate or align the selection and use of insecticides between sectors. In Sri Lanka, malathion and most synthetic pyrethroids had been legally restricted for use in mosquito control, prohibiting their use in agriculture, intended to delay the development of insecticide resistance.

Pesticide regulatory control

Pesticide legislation, including basic provisions for registration, import, export, manufacturing, procurement, marketing, application, storage, disposal, and enforcement were in place in the selected countries. In four countries, legislation covered all pesticide groups, including those for use in agriculture, livestock, households, and public health. However, in Cambodia and Vietnam, legislation covered agricultural and livestock pesticides, but excluded household and public health pesticides. Consequently, in Cambodia, there was no legislation for household

and public health pesticides, while in Vietnam, the health ministry had issued a separate decree on household and public health pesticides.

A single pesticide registration authority covering all pesticide groups, and housed under the agricultural ministry, was in place in Bangladesh, Nepal, Oman, and Sri Lanka. Registrar offices evaluated the data of submitted products for approval of their sale, use and conditions of use, and were advised by a multi-stakeholder committee, in a process that was harmonized for all pesticides. In Oman, pesticide registration had been initiated in 2014, but was still in transition in 2019, whereby part of the pesticide products available on the market were yet to be registered. Pesticide registration offices had few technical staff in all selected countries, and accordingly, data on risk assessment were not locally generated but obtained from the manufacturer. Two countries had a policy for restricting pesticide products for specific uses. For example, Sri Lanka, restricted most pyrethroids and malathion to public health and carbamates to agriculture.

In Cambodia and Vietnam, the central authority for pesticide registration covered only agricultural and livestock pesticides. In Cambodia, there was no separate registration for public health pesticides. In Vietnam, a separate registration scheme for household and public health pesticides had been established under the health ministry but there was little coordination or harmonization on pesticide registration procedures or standards between the agricultural and health ministries. Non-harmonized pesticide registration may compromise the quality of standards and norms and cause inconsistencies with respect to trade, sale, and use. Hence, a single registration authority for all pesticide groups has advantages (Table 2).

Compliance monitoring and enforcement of pesticide legislation was weak, reportedly with illegal pesticide products widely available in all countries. Moreover, pesticide quality control was a major challenge in all countries. Four countries had reference laboratories for pesticide analysis, but these laboratories were rarely used for analyzing vector control insecticides, whilst

their analyses comprised identification of active ingredient but excluded chemical-physical properties and relevant impurities.

Pesticide procurement

Methods of procurement should safeguard the timely availability of vector control insecticide products of high quality, in appropriate amounts, and with the intended effect on vector populations or pathogen transmission. Hence, pesticide procurement will benefit from having central coordination and guidelines. However, national guidelines on the methods and procedures of procurement of vector control insecticides were absent from five of the selected countries. All countries required that centralized procurements be restricted to WHO-prequalified products [31], but seldomly were the procured consignments of vector control insecticides submitted to quality control.

Malaria- and kala-azar elimination programs made centralized procurements of insecticides, commonly through a specialized procurement unit within the health ministry. However, procurements for the control of dengue and nuisance mosquitoes were primarily carried out by districts or municipalities, without coordination or harmonization between them, and using local funds. National health authorities had little or no control over the process, products or amounts of procurements by districts or municipalities. This lack of control was expressed as a concern because insecticide selection should be consistent with efforts to manage insecticide resistance, and because uncoordinated procurements could lead to accumulation of expired pesticides (Table 2).

Application methods and safety measures The malaria and kala-azar programs..

The malaria programs and kala-azar programs in the selected countries were supported by national guidelines and standard protocols on insecticide application, including for insecticide mixing, spraying, clean-up, and monitoring. However, the guidelines, protocols and,

Add references of the programs - line 433, 436.

accordingly, the training curricula, apparently needed updating in at least two countries; for example, it was reported that the quality of training on indoor residual spraying did not meet current international standards. With respect to dengue control, national guidelines and standard protocols on insecticide application were absent from three countries, while no national guidelines were known to be present for control of nuisance mosquitoes (Table 2).

Safety precautions to protect vector control spray workers was a critical shortcoming in programs all six countries. National guidelines or standard protocols for use of personal protective equipment, including gloves, mask, apron, boots, goggles [32], were either absent (4 countries), outdated (1 country) or under development (1 country). Basic training on the use of personal protective equipment by spray workers was commonly in place, but poor availability of quality personal protective equipment was a pressing problem across countries. In dengue programs, and in malaria programs where vector control operations were delegated to local actors, the provision of personal protective equipment for spray teams had to come from local budgets. However, a variable quality and provision of personal protective equipment between districts or municipalities were reported from four countries. We observed in two countries spray workers conducting routine space spraying operations with minimal protection. For space spraying, where droplets are small enough to be inhaled, workers need respiratory protective equipment [33], but in none of the countries, respirators were part of the personal protective equipment in space spraying operations.

A national scheme for health monitoring of vector control spray workers to detect pesticide poisoning (e.g. through blood tests or general health checks for signs and symptoms) was absent in all countries. For example, vector control spray workers in Sri Lanka were advised to get themselves checked regularly, but this was not standardized nor regulated. Moreover, a mechanism for compensation for pesticide poisoning among vector control spray workers was reportedly absent from all countries.

Pesticide storage and disposal

Insecticides procured by malaria- and kala-azar-elimination programs were stored centrally before being distributed to the provinces and districts for local storage to supply of spray operations on the ground. In addition, in Cambodia, Vietnam and Sri Lanka, the central stores were used for storing part of the insecticides for dengue control. These central stores were generally reported to be secure and safe for storing pesticides, but we were not able to verify these claims. In one country it was reported that these stores dated back from the first malaria eradication era and were not adequately maintained or modernized. Storage facilities at the provincial, district or municipal level were reportedly of questionable standard in all countries, commonly without a room purpose-made for storing pesticides. Of special concern were dengue control insecticides procured and stored by the districts or municipalities, without involvement or monitoring of the central level (Table 2). In Nepal there was a problem with gradual accumulation of expired pesticides at the central and provincial warehouses, suggesting inadequate coordination on stock management and procurement. Other countries mentioned there was no contemporary problem with accumulation of obsolete vector control insecticides.

None of the countries had a policy or national guideline in place for the safe and environmentally sound disposal of obsolete pesticides and pesticide waste, nor was disposal included as topic in the training for spray teams. In some vector control programs, empty pesticide containers were reportedly left in the field or where punctured to avoid reuse. In other programs, used containers were returned to the storage facilities, but were subsequently buried or burned. There was no facility for high-temperature incineration of pesticide waste in any of the countries. Several past efforts were made by countries, with external donor support, to centralize, safeguard and ship pesticide waste for incineration overseas. Insecticide-treated bed net products were redistributed in countries after several years of use by householders. However, none of the countries had a policy or plan in place for withdrawal and sound disposal of used nets.

Discussion

Why you are not discussing about leishmaniasis? only dengue and malaria.

Amidst changing epidemiological conditions - notably the elimination and emergence of vector-borne diseases [34, 35], countries were confronted with critical shortcomings in the procurement, application, safety measures, storage and disposal of vector control insecticides. These deficiencies in the pesticide 'lifecycle' have implications for the efficiency, effectiveness, and safety of vector control and, thus, for the control and elimination of vector-borne diseases. Vector control was used in centralized programs on malaria and visceral leishmaniasis and, in parallel, in decentralized programs on dengue and other arboviral diseases. The comparison between malaria programs and dengue programs provided lessons learned regarding the management of insecticides.

Central-level norms, such as national strategies, guidelines and standards, are vital to the quality and safety of insecticide use for all vector control programs [36], especially at decentralized levels where entomological expertise is scarce or absent [17, 37]. These norms provided direction and control at all levels in the malaria programs in our study, but were largely lacking from dengue programs, where practices of procurement, application, safety, storage, and disposal were variable between districts. A consequence of non-harmonized methods is that the quality and safety of operations are compromised. In turn, decentralized programs were better at facilitating the participation of multiple stakeholders and local communities in disease prevention activities and securing vector control financing from local budgets – aspects that centralized malaria programs have struggled with [38, 39].

Hence, an optimal vector control program, irrespective of the disease targeted, would have a combination of centralized and decentralized components, using harmonized guidelines and quality control measures while also engaging local stakeholders and communities in the prevention of disease. In this respect, the decentralized dengue program in Vietnam enjoyed support for national guidelines, standards, and training. Similarly, in Sri Lanka, the

implementation of vector control for prevention-of-reintroduction of malaria was largely decentralized to the districts and regions, whereas monthly coordination meetings at national level with district-level participation ensured harmonization on risk assessment and insecticide resistance management. The Sri Lankan example suggests how programs that achieved malaria elimination could remain vigilant after donor support has waned by establishing vector control capacity at district and regional level. Nonetheless, national leadership must continue to ensure that adequate resources are allocated to the prevention-of-reintroduction of malaria into the country [40, 41], rather than relying exclusively on priority setting at the district level.

Despite the potential synergies between malaria programs and dengue programs, in most instances there was little communication or collaboration between the two programs in individual countries, in terms of sharing of infrastructure, information, expertise and human resources. As entomological capacity was concentrated in malaria programs at central level, the dengue programs were largely missing out on expertise needed for their vector control decisions, whilst the demand for entomologists was not met by tertiary education systems. Therefore, firmer linkages should be established between programs, for optimized use of the limited entomological resources available in countries [37].

An area in which coordination is essential is insecticide resistance management [18, 42]. Countries in our study established monitoring systems for insecticide resistance in malaria vectors, often with external donor support, but these systems were rarely used for dengue vectors. Moreover, strategies for insecticide resistance management, where present, did not extend across programs or sectors, except in Sri Lanka. Notably, the routine and excessive use of insecticides against nuisance mosquitoes undermined the efficacy of dengue vector control in urban areas in two countries. Another challenge is that insecticide resistance management within programs was hindered by the scarcity of alternative options with unique modes of action [43]. Several novel vector control products have recently been prequalified by WHO [31].

However, in many elimination settings, only small amounts of insecticides are needed to target remaining transmission foci. The registration and shipment for minor-use products is generally unattractive to pesticide suppliers unless countries join forces on the registration and procurement of these products.

Another subject that warrants special attention is space spraying by districts and municipalities for control of dengue and nuisance mosquitoes. Worldwide, space spraying has been the mainstay response to dengue outbreaks for the past fifty years, despite its critiques asserting it has no impact [44, 45]. The research community has paid limited attention to space spraying and, to date, there is no epidemiological evidence that supports the use of space spraying for dengue control [46-51]. In our study, local health authorities continued to use space spraying for dengue control in the absence of evidence on its impact because alternative options for outbreak response were lacking. Also, a main driving force behind the continued use of space spraying was the demand from the public, because the highly visible operations provided the public with a sense of security against diseases and pests. Current international guidelines recommend space spraying for dengue control only in emergency situations to suppress an ongoing epidemic or to prevent an incipient one [52]. These norms were interpreted in several ways among our study countries, ranging from the restricted use in defined clusters based on efficient case reporting, to the extensive use where dengue cases had spread or where case reporting was deficient. Districts and municipalities continued using those insecticides against which vectors had developed high levels of resistance, and space spraying was mostly conducted outdoors, out of contact with the predominantly indoor-resting *Ae. aegypti* vector. Consequently, space spraying probably had little or no impact on vector populations, let alone on dengue incidence, which implies that valuable resources were wasted [53]. These insights call for a revision of international guidelines to help countries decide about the conditions under which the use of space spraying for dengue control is justified. Guidelines should take into account the 'external costs' of pesticide use [54], including health effects on spray workers in

situations where personal protective equipment and health monitoring are inadequate, and the adverse effects on non-target organisms [55, 56].

Strengths of our study were that it captured a combination of technical, organizational, and institutional aspects of vector control within countries, thus, offering insight into the contemporary challenges and opportunities. Also, the sample of six countries provided an array of parallels and divergences between countries, enabling us to extract lessons learned. We showed that the selected countries had slightly-poorer conditions of vector control insecticide management than other countries in their regions, suggesting that our findings are moderately generalizable for Asia and the Middle East, but the generalizability to other parts of the World may be limited. Another limitation of our study was that we acquired much of the information through verbal communication with respondents in each country, while opportunities for independent verification of conditions on the ground were limited.

Can structural improvements be made in the management of vector control insecticides? In the past decade, global policy initiatives on integrated vector management and pesticide management have been frustrated by slow progress at country level [1, 23, 38, 57]. Apparently, the gap between international policy, which called for multifaceted integrated approaches, and the reality on the ground has been too large. Funding support to advance the cause for integrated vector management and pesticide management has been meagre and, disturbingly, has not been substantially raised since WHO launched the Global Vector Control Response in 2017. Where available, international support contributed significantly through a ‘technocratic approach’, particularly through the establishment of capacity for insecticide resistance monitoring in malaria-endemic countries [23]. However, organizational or institutional aspects have received little attention at country, regional and global level. For example, despite insecticide resistance monitoring being in place, the effective management of insecticide resistance has been obstructed by weak intra- and intersectoral collaboration.

To break the impasse in transitioning towards integrated vector management, we propose that countries start from the bottom up by tackling how the contemporary management of vector control insecticides can be improved. A process of situation analysis, as conducted in our study, necessarily involves technical as well as organizational and institutional aspects. If followed through, this process leads to the required intra- and intersectoral linkages and new options for vector control, as has been envisaged in the Global Vector Control Response. Previous initiatives on integrated vector management were mostly centered around malaria control, however, dengue control can be a more viable entry point for establishing multi-partner participation, because of its decentralized management structure [38, 58]. In any case, international support is necessary to assist countries in situation analysis and action planning on vector control insecticide management. Moreover, follow-up technical assistance will be required, as indicated in our study, to support the development of national guidelines and standards on procurement, application methods, safety measures, storage, and disposal, and to facilitate coordination between programs and sectors.

Supporting information

S1 Table. Panel of experts consulted in the selected countries

Acknowledgments

All persons participating in the stakeholder meetings and interviews in the respective countries are gratefully acknowledged for their valuable inputs. Special thanks are due to Mannan Bengali (deceased), Sabera Sultana, Mya Sapal Ngon, Nguyen Thi Lien Huong, Tran Ahn Dung, Vu Nhat Linh, Dai Tran Cong, Subhash Lakhe, Lungten Wangchuk, Usha Kiran, Manjula Danansuriya, Manonath Marasinghe, Jayakody A. Sumith, Osama Ahmed, Vibol Chan,

Luciano Tuseo, Say Chy, and Jean Olivier Guintran, for their inputs and their support in facilitating the meetings during the country missions.

This paper's content is solely the responsibility of the authors and does not necessarily represent the official views of their respective organizations.

References

1. WHO. Global vector control response 2017-2030. Geneva: World Health Organization; 2017.

2. Kyu HH, Abate D, Abate KH, Abay SM, Abbafati C, Abbasi N, et al. Global, regional, and national disability-adjusted life-years (DALYs) for 359 diseases and injuries and healthy life expectancy (HALE) for 195 countries and territories, 1990–2017: a systematic analysis for the Global Burden of Disease Study 2017. The Lancet. 2018;392(10159):1859-922.

3. Golding N, Wilson AL, Moyes CL, Cano J, Pigott DM, Velayudhan R, et al. Integrating vector control across diseases. BMC medicine. 2015;13(1):249.

4. Wilson AL, Courtenay O, Kelly-Hope LA, Scott TW, Takken W, Torr SJ, et al. The importance of vector control for the control and elimination of vector-borne diseases. PLoS Neglected Tropical Diseases. 2020;14(1):e0007831.

5. Townson H, Nathan MB, Zaim M, Guillet P, Manga L, Bos R, et al. Exploiting the potential of vector control for disease prevention. Bull World Health Organ. 2005;83(12):942-7. PubMed Central PMCID: PMCEnglish.

6. Hemingway J. The role of vector control in stopping the transmission of malaria: threats and opportunities. Philosophical Transactions of the Royal Society B: Biological Sciences. 2014;369(1645):20130431.

7. Roiz D, Wilson AL, Scott TW, Fonseca DM, Jourdain F, Müller P, et al. Integrated *Aedes* management for the control of *Aedes*-borne diseases. PLoS Neglected Tropical Diseases. 2018;12(12):e0006845.

8. Bhatt S, Weiss DJ, Cameron E, Bisanzio D, Mappin B, Dalrymple U, et al. The effect of malaria control on *Plasmodium falciparum* in Africa between 2000 and 2015. Nature. 2015;526(7572):207-11.

9. van den Berg H, Zaim M, Yadav RS, Soares A, Ameneshewa B, Mnzava A, et al. Global trends in the use of insecticides to control vector-borne diseases. Environ Health Perspect. 2012;120(4):577-82.

10. Milliner J. The net mapping project. Washington, DC: USAID/President's Malaria Initiative; 2010.

11. WHO. Pesticides and their application for the control of vectors and pests of public health importance. WHO/CDS/NTD/WHOPES/ GCDPP/2006.1. Available: http://whqlibdoc.who.int/hq/2006/WHO_CDS_NTD_WHOPES_GCDPP_2006.1_eng.pdf Geneva: World Health Organization; 2006.

12. WHO. World malaria report 2019. Geneva: World Health Organization; 2019.

13. Killeen GF, Tatarsky A, Diabate A, Chaccour CJ, Marshall JM, Okumu FO, et al. Developing an expanded vector control toolbox for malaria elimination. BMJ Global Health. 2017;2(2):e000211.

14. Takken W, Knols BGJ. Malaria vector control: current and future strategies. Trends in Parasitology. 2009;25:101-4.

15. Thomas MB, Godfray HCJ, Read AF, van den Berg H, Tabashnik BE, van Lenteren JC, et al. Lessons from agriculture for the sustainable management of malaria vectors. PloS Medicine. 2012;9(7):e1001262.

16. Devine GJ, Overgaard HJ, Paul RE. Global vector control guidelines–the need for co-creation. Trends in Parasitology. 2019;35(4):267-70.

17. Horstick O, Runge-Ranzinger S, Nathan MB, Kroeger A. Dengue vector-control services: how do they work? A systematic literature review and country case studies. Trans Royal Soc Trop Med Hyg. 2010;104:379-86.

18. Mnzava AP, Knox TB, Temu EA, Trett A, Fornadel C, Hemingway J, et al. Implementation of the global plan for insecticide resistance management in malaria vectors: progress, challenges and the way forward. Malaria Journal. 2015;14(1):173.

19. Mnzava AP, Macdonald MB, Knox TB, Temu EA, Shiff CJ. Malaria vector control at crossroads: public health entomology and the drive to elimination. Transactions of The Royal Society of Tropical Medicine and Hygiene. 2014;108(9):550-4.

20. Alonso P, Engels D, Reeder J. Renewed push to strengthen vector control globally. Lancet. 2017;389(10086):2270-1.

21. WHO. Handbook for integrated vector management. WHO/HTM/NTD/VEM/2012.3. Available: http://whqlibdoc.who.int/publications/2012/9789241502801_eng.pdf. Geneva: World Health Organization; 2012.

22. WHO. Global strategic framework for Integrated Vector Management. WHO document WHO/CDS/CPE/PVC/2004.10. Geneva: World Health Organization; 2004.

23. van den Berg H, Gu B, Grenier B, Kohlschmid E, Al-Eryani S, da Silva Bezerra HS, et al. Pesticide lifecycle management in agriculture and public health: Where are the gaps? Science of the Total Environment. 2020;742:140598.

24. Najera JA, Gonzalez-Silva M, Alonso PL. Some lessons for the future from the global malaria eradication programme (1955–1969). PloS Med. 2011;8:e1000412.

25. WHO. Framework for a National Vector Control Needs Assessment. Geneva: World Health Organization; 2017.

26. IRAC. IRAC mode of action classification scheme. Version 9.4: Insecticide Resistance Action Committee, CropLife International; 2020.

27. Amelia-Yap ZH, Chen CD, Sofian-Azirun M, Low VL. Pyrethroid resistance in the dengue vector *Aedes aegypti* in Southeast Asia: present situation and prospects for management. Parasites & Vectors. 2018;11(1):332.

28. Al-Amin HM, Johora FT, Irish SR, Hossainey MRH, Vizcaino L, Paul KK, et al. Insecticide resistance status of *Aedes aegypti* in Bangladesh. bioRxiv. 2020.

29. Fernando HSD, Saavedra-Rodriguez K, Perera R, Black WC, De Silva BNK. Resistance to commonly used insecticides and underlying mechanisms of resistance in *Aedes aegypti* (L.) from Sri Lanka. Parasites & Vectors. 2020;13(1):1-14.

30. Boyer S, Lopes S, Prasetyo D, Hustedt J, Sarady AS, Doum D, et al. Resistance of *Aedes aegypti* (Diptera: Culicidae) Populations to Deltamethrin, Permethrin, and Temephos in Cambodia. Asia Pacific Journal of Public Health. 2018;30(2):158-66.

31. WHO. Prequalification Vector Control. http://www.who.int/pq-vector-control/en/ 2020.

32. WHO. Guidelines for personal protection when handling and applying pesticides: International Code of Conduct on Pesticide Management. Rome and Geneva: Food and Agriculture Organization and World Health Organization; 2020.

33. WHO. Space spray application of insecticides for vector and public health pest control: a practitioner's guide. http://whqlibdoc.who.int/hq/2003/WHO_CDS_WHOPES_GCDPP_2003.5.pdf. Geneva: World Health Organization; 2003.

34. Cotter C, Sturrock HJ, Hsiang MS, Liu J, Phillips AA, Hwang J, et al. The changing epidemiology of malaria elimination: new strategies for new challenges. The Lancet. 2013;382(9895):900-11.

35. Bhatia R, Dash AP, Sunyoto T. Changing epidemiology of dengue in South-East Asia. WHO South-East Asia Journal of Public Health. 2013;2(1):23.

36. van den Berg H, Yadav RS, Zaim M. Setting international standards for the management of public health pesticides. PloS Medicine. 2015;12(5):e1001824.

37. Nagpal BN, Knox TB, Risintha P, Yadav RS, Ghosh SK, Uragayala S, et al. Strengthening of vector control in South-East Asia: Outcomes from a WHO regional workshop. Journal of Vector Borne Diseases. 2018;55(4):247.

38. Chanda E, Ameneshewa B, Bagayoko M, Govere JM, Macdonald MB. Harnessing integrated vector management for enhanced disease prevention. Trends in Parasitology. 2017;33(1):30-41.

39. Atkinson JA, Vallely A, Fitzgerald L, Whittaker M, Tanner M. The architecture and effect of participation: a systematic review of community participation for communicable disease control and elimination. Implications for malaria elimination. Malaria Journal. 2011;10:225.

40. Shretta R, Baral R, Avanceña AL, Fox K, Dannoruwa AP, Jayanetti R, et al. An investment case to prevent the reintroduction of malaria in Sri Lanka. The American Journal of Tropical Medicine and Hygiene. 2017;96(3):602-15.

41. Tatarsky A, Aboobakar S, Cohen JM, Gopee N, Bheecarry A, Moonasar D, et al. Preventing the reintroduction of malaria in Mauritius: a programmatic and financial assessment. PLoS ONE. 2011;6(9):e23832.

42. Chanda E, Thomsen EK, Musapa M, Kamuliwo M, Brogdon WG, Norris DE, et al. An operational framework for insecticide resistance management planning. Emerging Infectious Diseases. 2016;22(5):773.

43. Hemingway J, Ranson H, Magill A, Kolaczinski J, Fornadel C, Gimnig J, et al. Averting a malaria disaster: will insecticide resistance derail malaria control? Lancet. 2016;387(10029):1785-8.

44. Gubler DJ. *Aedes aegypti* and *Aedes aegypti*-borne disease control in the 1990s: top down or bottom up. American Journal of Tropical Medicine and Hygiene. 1989;40:571-8.

45. Gubler DJ. Prevention and control of Aedes aegypti-borne diseases: lesson learned from past successes and failures. AsPac J Mol Biol Biotechnol. 2011;19(3):111-4.

46. Erlanger TE, Keiser J, Utzinger J. Effect of dengue vector control interventions on entomological parameters in developing countries: a systematic review and meta- analysis. Medical and Veterinary Entomology. 2008;22(3):203-21.

47. Esu E, Lenhart A, Smith L, Horstick O. Effectiveness of peridomestic space spraying with insecticide on dengue transmission; systematic review. Tropical Medicine & International Health. 2010;15(5):619-31.

48. Pilger D, De Maesschalck M, Horstick O, San Martin JL. Dengue outbreak response: documented effective interventions and evidence gaps. TropIKAnet. 2010;1(1):1-31.

49. Bowman LR, Donegan S, McCall PJ. Is dengue vector control deficient in effectiveness or evidence?: systematic review and meta-analysis. PLOS Negl Trop Dis. 2016;10(3):e0004551.

50. Newton EA, Reiter P. A model of the transmission of dengue fever with an evaluation of the impact of ultra-low volume (ULV) insecticide applications on dengue epidemics. The American Journal of Tropical Medicine and Hygiene. 1992;47(6):709-20.

51. Koenraadt C, Aldstadt J, Kijchalao U, Kengluecha A, Jones J, Scott T. Spatial and temporal patterns in the recovery of *Aedes aegypti* (Diptera: Culicidae) populations after insecticide treatment. Journal of Medical Entomology. 2007;44(1):65-71.

52. WHO. Dengue: guidelines for diagnosis, treatment, prevention and control. Available: http://apps.who.int/tdr/svc/publications/training-guideline-publications/dengue-diagnosis-treatment. Geneva: World Health Organization and the Special Programme for Research and Training in Tropical Diseases 2009.

53. Fernandes JN, Moise IK, Maranto GL, Beier JC. Revamping mosquito-borne disease control to tackle future threats. Trends in Parasitology. 2018;34(5):359-68.

54. Pretty J, Waibel H. Paying the price: the full cost of pesticides. In: Pretty J, editor. The Pesticide Detox: Towards a More Sustainable Agriculture. London: Earthscan; 2005. p. 39-54.

55. Abeyasuriya KTN, Nugapola NNP, Perera MDB, Karunaratne WIP, Karunaratne SP. Effect of dengue mosquito control insecticide thermal fogging on non-target insects. International Journal of Tropical Insect Science. 2017;37(1):11-8.

56. Bonds JAS. Ultra- low- volume space sprays in mosquito control: a critical review. Medical and Veterinary Entomology. 2012;26(2):121-30.

57. WHO. Sixty-third World Health Assembly, Geneva 17-21 May 2010, Resolutions and decisions. WHA63/2010/REC/1. Geneva: World Health Organization; 2010.

58. van den Berg H, Velayudhan R, Ebol A, Catbagan BHG, Turingan R, Tuso M, et al. Operational efficiency and sustainability of vector control of malaria and dengue: descriptive case studies from the Philippines. Malaria Journal. 2012;11(1):269.

Supplemental file

Click here to access/download
Supporting Information
Supplemental file.docx
