# Supplementary material for: Management of insecticides for use in disease vector control: Lessons from six countries in Asia and the Middle East
Source: PLoS Negl Trop Dis. 2021 Apr 30;15(4):e0009358. doi: 10.1371/journal.pntd.0009358 (PMC8115796; doi:10.1371/journal.pntd.0009358)
Supplement: Attachment — Submitted filename: SciRep_DRforMosquito2.docx [file pntd.0009358.s003.docx]

PLOS Neglected Tropical Diseases (research)

PNTD-D-20-01641

**Management of insecticides for use in disease vector control: Lessons from 4 six countries in Asia and the Middle East**

**General:**

This is timely and extremely important study wherein operational aspects related to Insecticidal use and management in VBD control/Elimination and Re-introduction of disease post elimination have been highlighted in 6 countries, Bangladesh, Cambodia, Nepal, Oman, Sri Lanka, and Vietnam.

The assessment covered technical, organizational, and institutional aspects of the following topics: pesticide regulatory control, pesticide procurement, insecticide application for vector control, insecticide resistance management, safety measures, pesticide storage, pesticide disposal, and waste management.

The study brought to the fore, disparities in the national guidelines and the manner in which the programme for the disease management is organised in these countries which had the bearing on insecticide use related policies and overall insecticide management.

Major VBDs addressed are Malaria, dengue and Kala Azar (In Nepal and Bangladesh).

The study highlights the advantages and disadvantages of decentralized and centralised procurements of insecticides and decision making.

Even though malaria has been eliminated in Sri Lanka, the malaria program intended to maintain its separate structure and capacity for vector control in the post-elimination scenario.

The number of entomologists ranged from zero to four per vector control program. Nepal had no entomologist was in place for any of the vector control programs on malaria, dengue or kala-azar, and local technicians were absent under the recently federalized government structure. In other countries, entomologists were mainly positioned within malaria programs, mostly at central offices and with assistance by technicians at sub-national or district level. In some countries, however, health authorities expressed concern about the continuity of entomological capacity once malaria has been eliminated.

In the light of this how WHO GVCR will be implemented. The ground reality of poor entomological capacity in the study countries amply justifies the role GVCR could play in enhancing capacity building and strengthening where implemented in letter and spirit.

**Methods:**

1. It is not clear what criteria was used to ensure representativeness of the study countries to other countries of the regions?

**Findings**

1. The study has been able to acquire qualitative information on how the national programmes on vector borne diseases are organised in the 6 study countries and the status of insecticide use, resistance, choices, disposal and management vis a vis other VC methods such as source reduction.
2. The reason for not undertaking susceptibility studies may be elucidated. Was this exclusively due to lack of manpower at the various levels of health sectors or lack of appreciation of the fact that this is one of the most important activities of the programme being a decision making tool.

**3. Limitations of the study: Should be mentioned in the MS**

1. Five countries in Asia seem representing the entire Asia rather well but only Oman in Middle East did not appear to represent the entire region. Therefore, findings in this country cannot be generalized for the entire middle east region.

The study should have mentioned about an ideal scenario or a model that aligns with GVCR concept and practical to practice.

2. By comparison, the six selected countries scored an average of 40% which was much lower, against 52% suggesting that the selected countries had slightly poorer conditions of vector control insecticide management than other countries in their regions.

3. The quantitative data on insecticide management should have been provided. For example, quantities used, frequency of use, target population coverage, susceptibility studies, intensity assays for resistance monitoring and decision making, etc. In the current manuscript this is lacking.

**Typographical Errors:**

1. Line No. 313: extend instead of extent
2. Line No. 318: Remove was
3. Lines 367-371: Re-phrase the sentence
