# Supplementary material for: Management of insecticides for use in disease vector control: Lessons from six countries in Asia and the Middle East
Source: PLoS Negl Trop Dis. 2021 Apr 30;15(4):e0009358. doi: 10.1371/journal.pntd.0009358 (PMC8115796; doi:10.1371/journal.pntd.0009358)
Supplement: Attachment — Submitted filename: Response to reviewers.docx [file pntd.0009358.s006.docx]

Dr Tiago Donatelli Serafim

Associate Editor

PLOS Neglected Tropical Diseases

9^th^ February 2021

Response letter: PLOS Neglected Tropical Diseases submission (PNTD-D-20-01641)

Dear Dr Serafim,

We have addressed the comments by the editor and the four reviewers (including those in two pdf files and a Word file that had been attached) and are happy to send you herewith the revised version of the manuscript.

We have added our responses to each of the reviewers’ comments below in **bold font**. The line numbers refer to those in the document with track-mode changes.

The quality of Figure 1 has been upgraded.

We look forward to your response.

Sincerely,

The authors

----------------------

Reviewer #1

Authors have well written regarding management of insecticides in selected countries. They could have mentioned in their discussion more about quality control during procurement.

**Response: We thank the reviewer for these compliments. Regarding procurement, we added on line 515-518 (Discussion section): “For example, central coordination over pesticide procurement has been lacking in decentralized dengue control programs, with implications for quality control, efficacy of interventions and insecticide resistance management.”**

In Integrated vector management, checking efficacy of vector control operations like Indoor residual spray is very important to assess the role of vectors and their transmission. Correlation between entomological and epidemiological studies plays an important role in elimination, control, and re-introduction of disease. These studies have to be performed in all countries where vector control and epidemiological interventions are used. As mentioned, technical expertise is required. Entomology experts must be recruited at grass root level and national level also for proper guidance, vector identification and monitoring. This will also help in developing new vector control measures which can be introduced in Integrated vector management. Technical expertise can bridge the gap between international policy and bureaucracy in respective countries. Mainly national experts must play an important role in quality control, identification of resistance and assessing the efficacy of vector control used. Insecticide selection, rotation and mosaic usage has to be suggested according to the current requirement without any delays, which helps in better evaluation, management of disease outbreaks and transmission. The authors should have more focused on the above mentioned which will help in designing global vector control practices. The lessons learned for the studies in different countries should be used in an effective manner for prevention of endemicity in some countries.

**Response: The reviewer suggests we pay more attention to the need for entomological expertise in the Discussion. In response, we added the reviewer’s inputs, in summarized form, to the Discussion section at the appropriate place (line 542-544): “Entomological expertise and capacity are essential for entomological surveillance, monitoring and management of insecticide resistance, and for studying efficacy and effectiveness of existing and new interventions.”**

----------------------

Reviewer #2:

This manuscript is timely and of high significance for formulating international/global policy for insecticide use management in the countries of different regions of WHO.

----------------------

Reviewer #3:

The study titled "Management of insecticides for use in disease vector control: Lessons from six countries in Asia and the Middle East" gathered data from six countries with the goal to to assessed the effectivity of current vector control interventions. The authors showed the shortcomings in current vector control programs in these six countries, and the need for the proper application of current vector control interventions.

The authors should discuss more about leishmaniasis programs and not only dengue and malaria programs, this would strengthen the main goal of the manuscript. The manuscript is well written, however, some references are missing and this needs to be corrected.

**Response: We have increased the number of times we referred to leishmaniasis in our Discussion, even though we had less information available on leishmaniasis than for malaria or dengue, as we explain in response to this reviewer’s final comment. Moreover, we added “visceral leishmaniasis” in line 307.**

**Missing references have been added (see our responses to the additional comments below).**

**For consistency, we replaced “kala-azar” with “****visceral leishmaniasis” throughout the text.**

Additional comments by reviewer in pdf file (PNTD-D-20-01641_reviewed.pdf) that had been attached:

Line 50-55 You are referring from line 50 to 55 about malaria and dengue only, what about leishmaniasis programs? There are plenty of reports stating that the effect of insecticides were not as effective due to lack of proper training for the application of insecticides. Same comment applies for line 69 to 73. You should discuss as well leishmaniasis control programs when compared to dengue and malaria

**Response: We had less information available on leishmaniasis than for malaria or dengue, as we explain in response to this reviewer’s final comment. Nevertheless, we made modifications to lines 49-53 and to lines 72 by including “visceral leishmaniasis”.**

Line 95-96- Changed to the following: Their strategy is not disease-specific but seeks to .... (as it sounds repetitive)

**Response: modified as suggested (L98)**

Line 95-96- Missing reference for line 98-102

**Response: reference added (line 104)**

Line 95-96- Missing reference for line 106-107

**Response: reference added (line 109)**

Line 123-124: Why was India not included as selected country in your study? India is highly endemic for Dengue, leishmaniasis and malaria and their major vector control program is through the use of insecticides. I think it would bring impact to the manuscript to at add or discussed the current situation there.

**Response: We agree India would also have been a good country for conducting the study. However,** **as described in the Methods section, country selection was foremost based on country interest in situation analysis and action planning on public health pesticide management, as communicated to WHO Regional Offices. No change made.**

Line 178. The six countries had..

**Response: modification made as suggested (L188)**

Line 183-184. Missing references of pyrethroid resistance and outdoor-bitting behavior of the vectors

**Response: two new references have been added (L194)**

Add references on the actors column in Table 1 for each of the vector control programs

**Response: the information in Table 1 was collected first-hand through our country visits, not from the literature. Moreover, most of these programs do not have a dedicated website or program publication. No change needed.**

431: The malaria and kala-azar programs

**Response: modification made as suggested (L446)**

Add references of the programs - line 433, 436.

**Response: As mentioned above, most of these programs do not have a dedicated website or program publication. No change needed.**

L485 (Discussion). Why you are not discussing about leishmaniasis? only dengue and malaria

**Response: We took malaria as example of centralized programs and dengue as example of decentralized programs. There was much less information available on leishmaniasis programs, which were existing in only 2 countries. Hence, our emphasis in the Discussion was on malaria and dengue. To address this point, we added to line 508 (underlined): ”The comparison between malaria programs and dengue programs, in particular, provided lessons learned regarding the management of insecticides.”
Nevertheless, we added “visceral leishmaniasis programs” on line 513 and also on line 540.**

----------------------

Reviewer #4:

The manuscript by Dr. Yadav et al. brings an extremely relevant assessment of some characteristics of vector control programmes in six different countries. It brings an intersting perspective and raises fundamental issues to the improvement of similar national initiatives across the globe. It is very well designed, conducted, written. I have only very minor text edits (see file).

**Response: We thank the reviewer for these compliments.**

Additional comments by reviewer in Word file (SciRep_DRforMosquito2.docx) that had been attached:

This is timely and extremely important study wherein operational aspects related to Insecticidal use and management in VBD control/Elimination and Re-introduction of disease post elimination have been highlighted in 6 countries, Bangladesh, Cambodia, Nepal, Oman, Sri Lanka, and Vietnam. The assessment covered technical, organizational, and institutional aspects of the following topics: pesticide regulatory control, pesticide procurement, insecticide application for vector control, insecticide resistance management, safety measures, pesticide storage, pesticide disposal, and waste management. The study brought to the fore, disparities in the national guidelines and the manner in which the programme for the disease management is organised in these countries which had the bearing on insecticide use related policies and overall insecticide management. Major VBDs addressed are Malaria, dengue and Kala Azar (In Nepal and Bangladesh). The study highlights the advantages and disadvantages of decentralized and centralised procurements of insecticides and decision making. Even though malaria has been eliminated in Sri Lanka, the malaria program intended to maintain its separate structure and capacity for vector control in the post-elimination scenario. The number of entomologists ranged from zero to four per vector control program. Nepal had no entomologist was in place for any of the vector control programs on malaria, dengue or kala-azar, and local technicians were absent under the recently federalized government structure. In other countries, entomologists were mainly positioned within malaria programs, mostly at central offices and with assistance by technicians at sub-national or district level. In some countries, however, health authorities expressed concern about the continuity of entomological capacity once malaria has been eliminated.

**Response: This is an accurate partial summary of the manuscript. No change needed.**

In the light of this how WHO GVCR will be implemented. The ground reality of poor entomological capacity in the study countries amply justifies the role GVCR could play in enhancing capacity building and strengthening where implemented in letter and spirit.

**Response: The reviewer suggests that entomological capacity should be enhanced. We added to the Discussion section (line 542-544): “Entomological expertise and capacity is essential for entomological surveillance, monitoring and management of insecticide resistance, and for studying efficacy and effectiveness of existing and new interventions.”**

Methods: It is not clear what criteria was used to ensure representativeness of the study countries to other countries of the regions?

**Response: As described in the Methods section, country selection was based on country interest in situation analysis and action planning on public health pesticide management, as communicated to WHO Regional Offices. We added as criterium (L125-126): “…we verified that interested countries had at least one vector-borne disease control program with a vector control component.” We did not use criteria related to insecticide management because, at that time, we did not know the situation at country level. Instead, we evaluated the representativeness of the countries only after the study was conducted, when results from a global survey became available (reference nr. 23). We added to/modified the text (underlined) (L133-136): “After completion of our data collection, we verified whether the selected countries were representative for their regions. This was possible through examination of the results from a separate global survey on pesticide management, which became available in 2020 [23].”**

Findings. The study has been able to acquire qualitative information on how the national programmes on vector borne diseases are organised in the 6 study countries and the status of insecticide use, resistance, choices, disposal and management vis a vis other VC methods such as source reduction.

**Response: This is an accurate partial summary of the manuscript. No change needed.**

The reason for not undertaking susceptibility studies may be elucidated. Was this exclusively due to lack of manpower at the various levels of health sectors or lack of appreciation of the fact that this is one of the most important activities of the programme being a decision making tool.

**Response: We added to line 369-370: “because of inadequate technical resources or because the importance of resistance monitoring was under-valued by programs.”**

3. Limitations of the study: Should be mentioned in the MS

**Response: Limitations have been mentioned in Lines 587-595. This section is slightly modified to highlight the limitations.**

1. Five countries in Asia seem representing the entire Asia rather well but only Oman in Middle East did not appear to represent the entire region. Therefore, findings in this country cannot be generalized for the entire middle east region.

**Response: Please see our response above to the same reviewer regarding the representativeness of our selected countries. We do not assert that the selected countries represent their entire region, nor does this appear from the paper’s title. However, we verified the degree of representativeness after completion of data collection, as described in the Methods section (L133-143).**

The study should have mentioned about an ideal scenario or a model that aligns with GVCR concept and practical to practice.

**Response: We do not wish to present an ideal model. Our purpose was not to have a pre-conceived notion on what would be ideal, but rather, to study the local situation to find out what worked or did not work, and why. As it turned out, it was often difficult to generalize between countries, although we distilled a number of general lessons learnt, as presented in Table 2.**

2. By comparison, the six selected countries scored an average of 40% which was much lower, against 52% suggesting that the selected countries had slightly poorer conditions of vector control insecticide management than other countries in their regions.

**Response: Yes, we described on lines 141-143: “This suggests that the selected countries for our study had slightly poorer conditions of vector control insecticide management than other countries in Asia and the Middle East.”**

3. The quantitative data on insecticide management should have been provided. For example, quantities used, frequency of use, target population coverage, susceptibility studies, intensity assays for resistance monitoring and decision making, etc. In the current manuscript this is lacking.

**Response: Since our study was a qualitative assessment of the local situation, its context, and the causes of deficiencies, it was beyond the scope of our study (as was agreed with the countries) to present detailed quantitative data on insecticide use and insecticide management.**

Typographical Errors:

Line No. 313: extend instead of extent

**Response: corrections were made as suggested (L325)**

Line No. 318: Remove was

**Response: corrections were made as suggested (L330)**

Lines 367-371: Re-phrase the sentence

**Response: Sentence was re-phrased (L382-384)**

----------------------

Additional file PNTD-D-20-01641_revised.pdf:

A few minor corrections were accepted and corrected in the manuscript

----------------------

Editor’s comments:

Points to be addressed:

1. Manuscript will benefit if tick born diseases are also mentioned.

**Response: In line 81, we added “and tick-borne diseases”. Line 86: we added “or tick [vectors]”.**

2. Line131: Score% is vague. Authors should detail more.

**Response: This section was rewritten for better clarity (L133-143)**

3. Line 354: "manage managing"?

**Response: changed to ‘management’ (L355)**

4. Lines 556-570: Authors should reinstate how they get to the conclusions stated on those sentences.

**Response: We presume the editor refers to L566-570 (not L556) in the previous version, which discusses the selection of countries for the study. These sentences have been modified for improved clarity (Line 587-595)**

----------------------

Reviewer's Responses to Questions

-Are the objectives of the study clearly articulated with a clear testable hypothesis stated?

-Is the study design appropriate to address the stated objectives?

-Is the population clearly described and appropriate for the hypothesis being tested?

-Is the sample size sufficient to ensure adequate power to address the hypothesis being tested?

-Were correct statistical analysis used to support conclusions?

-Are there concerns about ethical or regulatory requirements being met?

Reviewer #1: Yes, the objectives were stated properly.

Reviewer #2: Yes

Reviewer #3: Minor revisions.

Reviewer #4: (No Response)

**Response: No change needed**

----------------------

Results

-Does the analysis presented match the analysis plan?

-Are the results clearly and completely presented?

-Are the figures (Tables, Images) of sufficient quality for clarity?

Reviewer #1: Results are clear and presented current situations success and failures in vector and disease management

Reviewer #2: Yes

Reviewer #3: Minor revisions. Some references are missing in the tables. Please add them.

Reviewer #4: (No Response)

**Response: No change needed (see our responses above)**

----------------------

Conclusions

-Are the conclusions supported by the data presented?

-Are the limitations of analysis clearly described?

-Do the authors discuss how these data can be helpful to advance our understanding of the topic understudy?

-Is public health relevance addressed?

Reviewer #1: The authors could have suggested more conclusions which will help in better coordination at grass root level, state level, national and international level such as technical expertise role in entomology and epidemiology of diseases. They could suggested measures or bridging gaps of the above for elimination of disease and avoiding re introduction of diseases in respective countries.

Reviewer #2: It will be better if the authors conclude their findings in a succinct manner at the end of the discussion or separately mentioning the limitations

Reviewer #3: The discussion are focused mainly in Dengue and Malaria vector control but not leishmaniasis. Please discuss and contrast the lack of studies in leishmaniasis compared to Dengue and Malaria if needed.

Reviewer #4: (No Response)

**Response: We added a Conclusions paragraph at the end of the Discussion (L623-631): “In conclusion, the deficiencies in the management of the pesticide ‘lifecycle’, as described in this study, is a neglected subject that has important implications for the control and elimination of vector-borne diseases. Several lessons have been learnt on how to improve organization and coordination of vector control and insecticide management within and between programs and between sectors. Essential to all programs in which insecticides are used is that adequate entomological expertise is available and national-level guidelines and standards on how to manage insecticides are executed. Further efforts are needed to identify specific shortcomings in other countries and to establish an inclusive process of planning to make constructive improvements.”**

**We have addressed the issue of limitations in one of our responses above (see Discussion section). Also, we have addressed the issue related to leishmaniasis in our comments above.**

--------------------

Editorial and Data Presentation Modifications? Use this section for editorial suggestions as well as relatively minor modifications of existing data that would enhance clarity. If the only modifications needed are minor and/or editorial, you may wish to recommend “Minor Revision” or “Accept”.

Reviewer #1: (No Response)

Reviewer #2: Some minor edits necessary as indicated in the attached file

Reviewer #3: Minor revision

Reviewer #4: (No Response) -------------------- <

**Response: the edits by reviewer #2 have already been dealt with above (see Reviewer #2)**
